# Evaluating processes of care and outcomes of children in hospital (EPOCH): study protocol for a randomized controlled trial

**DOI:** 10.1186/s13063-015-0712-3

**Published:** 2015-06-02

**Authors:** Christopher S. Parshuram, Karen Dryden-Palmer, Catherine Farrell, Ronald Gottesman, Martin Gray, James S. Hutchison, Mark Helfaer, Elizabeth Hunt, Ari Joffe, Jacques Lacroix, Vinay Nadkarni, Patricia Parkin, David Wensley, Andrew R Willan, For the Canadian Critical Care Trials Group

**Affiliations:** Critical Care Program, Center for Safety Research, and Senior Scientist, Child Health Evaluative Sciences, The Research Institute, Hospital for Sick Children, Toronto, ON M5G 1X8 Canada; Department of Paediatrics, University of Toronto, Toronto, ON Canada; Critical Care Program, Center for Safety Research Hospital for Sick Children, Toronto, ON Canada; CHU-St. Justine, Montreal, QC Canada; Montreal Children’s Hospital, Montreal, QC Canada; St. George’s Hospital, Tooting, London, UK; Hospital for Sick Children, Toronto, ON Canada; Children’s Hospital of Philadelphia, Philadelphia, PA USA; The Johns Hopkins Hospital, Baltimore, MD USA; Stollery Children’s Hospital, Edmonton, AB Canada; BC Children’s Hospital, Vancouver, BC Canada

**Keywords:** Randomized controlled trial, Early warning system, Children, Cardiac arrest, Mortality, ICU

## Abstract

**Background:**

The prevention of near and actual cardiopulmonary arrest in hospitalized children is a patient safety imperative. Prevention is contingent upon the timely identification, referral and treatment of children who are deteriorating clinically. We designed and validated a documentation-based system of care to permit identification and referral as well as facilitate provision of timely treatment. We called it the Bedside Paediatric Early Warning System (BedsidePEWS). Here we describe the rationale for the design, intervention and outcomes of the study entitled Evaluating Processes and Outcomes of Children in Hospital (EPOCH).

**Methods/Design:**

EPOCH is a cluster-randomized trial of the BedsidePEWS. The unit of randomization is the participating hospital. Eligible hospitals have a Pediatric Intensive Care Unit (PICU), are anticipated to have organizational stability throughout the study, are not using a severity of illness score in hospital wards and are willing to be randomized. Patients are >37 weeks gestational age and <18 years and are hospitalized in inpatient ward areas during all or part of their hospital admission.

Randomization is to either BedsidePEWS or control (no severity of illness score) in a 1:1 ratio within two strata (<200, ≥200 hospital beds). All-cause hospital mortality is the selected primary outcome. It is objective, independent of do-not-resuscitate status and can be reliably measured. The secondary outcomes include (1) clinical outcomes: clinical deterioration, severity of illness at and during ICU admission, and potentially preventable cardiac arrest; (2) processes of care outcomes: immediate calls for assistance, hospital and ICU readmission, and perceptions of healthcare professionals; and (3) resource utilization: ICU days and use of ICU therapies.

**Discussion:**

Following funding by the Canadian Institutes of Health Research and local ethical approvals, site enrollment started in 2010 and was closed in February 2014. Patient enrollment is anticipated to be complete in July 2015. The results of EPOCH will strengthen the scientific basis for local, regional, provincial and national decision-making and for the recommendations of national and international bodies. If negative, the costs of hospital-wide implementation can be avoided. If positive, EPOCH will have provided a scientific justification for the major system-level changes required for implementation.

Trial registration: NCT01260831 ClinicalTrials.gov date: 14 December 2010.

**Electronic supplementary material:**

The online version of this article (doi:10.1186/s13063-015-0712-3) contains supplementary material, which is available to authorized users.

## Background

The Evaluation of Processes of care and the Outcomes of Children in Hospital (EPOCH) study is a cluster-randomized trial comparing the effect of the Bedside Paediatric Early Warning System (BedsidePEWS) with standard care. The outcomes are clinical, processes of care and resource utilization. Here we describe the rationale for the trial, the intervention and the outcomes used, conflict of interest management and the potential impact of the study.

### The problem and current treatments

Cardiopulmonary arrest occurs in 0.1-20/1000 children in hospital wards [1-4], with hospital survival of 27-50% [5-7]. Despite optimal resuscitation and post-arrest care, in-hospital cardiac arrest is associated with significant mortality and acquired morbidity in survivors [3,6,8-11], making prevention the best strategy [6,8,12-23].

### Prevention of near and actual cardiopulmonary arrest

Prevention of near and actual cardiopulmonary arrest in hospitalized children is a patient safety imperative. Prevention is contingent upon the timely identification, referral and treatment of children whose conditions are clinically deteriorating [24,25]. Preventative strategies have focused on the latter portion of this sequence, primarily through improved access to ICU expertise through deployment of Medical Emergency Teams-Rapid Response Teams (MET-RRT). However, studies addressing methods to identify those in need have been few and of limited quality [26].

### Emergency response teams

To date, MET-RRT have not fulfilled their promise. In adult inpatients, following several positive [27-29] and negative [30-33] single-center studies, a 23-hospital, cluster-randomized clinical trial compared implementation of un-validated calling criteria plus MET-RRT with standard care (no MET-RRT, no explicit calling criteria). The primary outcome was a composite of unexpected death, unplanned ICU admission and cardiac arrest. This negative trial [34] provides several important lessons: (1) calling criteria were met in <50% of patients with urgent ICU admission, suggesting that the criteria did not identify patients at risk. (2) The number of patients without unexpected death, unplanned ICU admission or cardiac arrest, who met one or more of these calling criteria is unknown (similar to pediatric studies) [35-39]. (3) Approximately 90% of the patients who met criteria were referred to ICU teams in both MET and control hospitals, suggesting that the calling criteria added little to the standard model.

In children, only before-and-after studies have been published [35-39], of which one is multi-center [40]. The two studies showing reduced all-cause hospital mortality were performed over 6 [36] and 7½ years [38], influenced the findings of a systematic review of pediatric MET-RRT [41], which include no RCTs, and reinforced the potential for description of time-related improvement [42,43] to be ascribed to MET-RRT. Furthermore, the additional benefit from MET-RRT without appropriate ‘afferent limb’ identification mechanisms may be questionable in hospitals where the efferent limb response-ICU expertise is readily available [25].

### Mechanisms to identify children at risk for near and actual cardiopulmonary arrest

There are eight published pediatric mechanisms to identify children at risk for cardiac arrest [1,35,37,44-47]. The development of most has been methodologically limited [48]. There are four published calling criteria [35,37,46,47]. The Cincinnati criteria include subjective elements and have been modified [35,49]. The Baltimore criteria are subjective and/or triggered by acute medical diagnoses [37]. The Bristol tool is subjective and uses thresholds for the airway (nebulized epinephrine, ‘tiring’) and disability [46]. The Melbourne criteria identified 10/24 (42%) patients who had cardiac arrests in their hospital [38] and similar proportions in other hospitals [40,50].

There are four published pediatric scores [1,44,45,48]. The Brighton score uses behavior, circulatory (color, relative tachycardia) and respiratory (relative tachypnea) domains and ‘persistent vomiting following surgery’ with a range of 0 –11 [44]. Evaluation of this score in a cohort of 2979 children in Cincinnati Children’s Hospital found a sensitivity of 78% and specificity of 82% [49]. In the development cohort, the Cardiff and Vale score had a sensitivity of 69% and specificity of 90%. Inspection of the score reveals that children in the first months of life may be expected to routinely score at or above the threshold (2 points for systolic blood pressure <70 mmHg and respiratory rate >50 breaths/min) [45].

### The BedsidePEWS

The BedsidePEWS score differentiated between children who were urgently admitted to PICU and ‘well’ hospitalized children, with at least 1 h notice with a sensitivity of 83% and specificity of 95% [48]. In a multi-center validation study of 2074 patients the score performed well in sub-populations [51]. Among ‘case patients’ the score increased over time leading up to an urgent PICU admission or code-blue event, and was independent of the number of risk factors for cardiac arrest. [51].

The score was reliably calculated with an intraclass correlation coefficient of 0.92 and was associated with statistically significant improvements in clinical and process of care outcomes [52]. The quality of development, validation and evidence of reliability supports the conclusion that the BedsidePEWS score is equivalent or superior to others and provides a rationale for its use as the intervention in EPOCH (Table 1).

**Table 1:** **Differences between BedsidePEWS and other severity of illness scores**

(1)	A validated severity of illness score that is better at identifying patients at risk than the retrospective opinion of frontline nurses
(2)	Complete integration of scoring into routine documentation
(3)	Explicit care recommendations derived from the opinions of 280 healthcare professionals
(4)	Nurse-educator developed, provider tested implementation program
(5)	Pilot evaluation showing improved outcomes without additional resources

The objectives of EPOCH are to evaluate the impact of the BedsidePEWS on early identification of children at risk for near and actual cardiopulmonary arrest, hospital mortality, processes of care and ICU resource utilization. We hypothesize that the BedsidePEWS improves early detection of critical illness, reduces mortality, improves processes of care and does not increase healthcare resource utilization.

## Methods

EPOCH is a cluster-randomized trial comparing the BedsidePEWS with standard care (no severity of illness score) in hospitals with a pediatric intensive care unit (ICU). The unit of randomization is the participating hospital. Eligible hospitals provide care for more than a total of 200 eligible inpatient admissions in eligible inpatient wards each year, have specialized pediatric physicians and have at least one PICU. Eligible inpatient wards are defined as areas where care is provided to children who are admitted to the hospital, other than the NICU, PICU, operating rooms and other designated areas where anesthetist-supervised procedures are performed. Emergency departments that care for admitted patients may be regarded as an eligible ‘ward’ (Table 2). Eligible patients are >37 weeks gestational age and <18 years, and they have received care in an eligible inpatient ward. To ensure that major system changes do not introduce bias, we will exclude hospitals that plan to introduce or discontinue an MET-RRT during the study, are already using a severity of illness score in wards or consider randomization unacceptable. Hospitals with a pre-existing MET-RRT are eligible to participate.

**Table 2:** **Study definitions**

Eligible inpatient wards	Areas where care is provided to patients who are admitted to the hospital, other than the PICU, NICU, operating rooms and other designated areas where anesthetist-supervised procedures are performed
	Admitted patients cared for in emergency departments will be regarded as in an eligible ‘ward’ if the documentation format is the same in the emergency department as in the inpatient ward. If the emergency department continues to use a separate ‘emergency department’ documentation record for admitted patients then the emergency department will be deemed an ineligible area
PICU	A PICU is defined as a designated staffed area for prolonged mechanical ventilation, invasive monitoring and circulatory support for children, including but not limited to neonates. Other areas designated for patients of increased acuity, such as ‘constant observation’ or ‘high dependency’ or ‘step-down’ units, will be regarded as part of the PICU where the PICU staff physicians are wholly or jointly responsible for the care of children in these areas (can write orders in the chart). Routinely admitted patients will include children beyond the neonatal period who are <12 years of age at admission
MET-RRT	An MET-RRT is defined as an identified team of one or more trained healthcare professionals who report to an on-service PICU physician and perform urgent consultations on hospital inpatients. An MET-RRT ‘call’ is analogous to an ICU consultation in hospitals without an MET-RRT. As the effectors of expertise, the impact of the MET-RRT (or other ICU team) is dependent upon appropriate identification of patients at risk and timely referral
Urgent PICU admission	An admission to the PICU with departure from the event location in <6 h from the time the PICU admission was initiated. Initiation is the time when the PICU admission is confirmed, or confirmed as a ‘definite possibility following surgery’ in cases where post-operative care in the PICU might be required. PICU admissions initiated in the OR are also regarded as urgent ICU admissions, irrespective of the time between initiation and departure from the OR
Time of transfer	Transfer is when a patient is transferred urgently to a pediatric intensive care unit (PICU) in the participating hospital. The time of transfer is the arrival in the PICU. When a patient is admitted urgently from an eligible hospital ward to a PICU via a procedure in the OR, the time of transfer to the ‘PICU’ is regarded as beginning at the time of departure from the inpatient ward to the operating room. Treatments other than cardiopulmonary resuscitation provided in the operating room are not included in the calculation of the clinical deterioration event. Unexpected events occurring in the operating room that require postoperative/post-anesthetic care in the PICU, in patients who were not anticipated to require PICU at the time the patient was transferred from the inpatient ward to the operating room, will not be regarded as clinical deterioration events
Study Day	Study days are calendar days; they begin at 00:00:00 and end at 23:59:59
Study weeks	begin on Monday and end on Sunday. The first week of the study is study week 01

### Intervention: BedsidePEWS

The BedsidePEWS documentation-based system of care replaces existing documentation systems for vital signs in inpatient ward areas in hospitals randomized to the intervention. There are four elements in the system: (1) the BedsidePEWS score; (2) the BedsidePEWS documentation record; (3) score-matched care recommendations, developed using the responses of 280 healthcare professionals (80 community, 200 referral) surveyed to determine ‘reasonable’ care in each of the domains of vital sign assessment, secondary review, continuous monitoring and ICU consultation, and customized for each implementation; (4) the education program, developed by two nurse educators to support implementation and maintain expertise, including the BedsidePEWS Instructor course and frontline education program.

### Randomization

Participating hospitals are randomized in a 1:1 ratio to intervention or control. Computerized randomization is balanced by the use of two strata of hospital size: <200 vs. ≥200 eligible inpatient ward beds. Allocation is concealed until the start of the study measurements and is revealed in the 2nd week after the start of data collection at each site (Additional file 1).

### Primary outcome: all-cause hospital mortality

The primary outcome is all-cause hospital mortality in eligible inpatients who were cared for in an eligible inpatient ward at some point during their hospital stay. This includes anticipated deaths in children with ‘do not resuscitate’ orders. Mortality events in children cared for exclusively in the PICU, NICU or emergency department, or combinations thereof, are excluded.

### Secondary outcomes: clinical

The main secondary outcome is the significant clinical deterioration event (SCDE), a composite outcome comprised of the treatment(s) provided or death prior to transfer from an inpatient ward. An SCDE is defined as the provision of significant pre-specified respiratory or circulatory therapies or cardiopulmonary resuscitation in the 12 h before transfer from the inpatient ward or during the hour after transfer, or death without a DNR order in an inpatient ward (Table 2).

EPOCH includes eight additional clinical outcomes (Table 3): (1) the nature of SCDE rated on the seven-point Children’s Resuscitation Intensity Scale (Table 4), which measures the timing of interventions in children for whom active resuscitation is anticipated; (2) potentially preventable cardiac arrest in patients experiencing a cardiac arrest while in an eligible inpatient ward. This excludes patients with a preceding DNR order. Potentially preventable is defined as the degree to which ‘events may have been avoided given the application of reasonable current (2011-13) standards of practice by an average practitioner and system anticipated to manage the condition in question’ [53-57]. Events with a consensus rating from two expert reviewers of ≥4/6 are defined as potentially preventable cardiac arrests (Table 5) [55,56]. (3) Unplanned hospital re-admission; (4) urgent PICU re-admission, both within 48 h of discharge. Finally, in all patients admitted urgently to the PICU from a hospital inpatient ward measurement was made of: (5) the predicted risk of mortality (PIM2 score) [58,59], (6) PICU mortality, (7) organ dysfunction (PELOD score) [60] and (8) ventilator-free days to 28 days. Urgent PICU admission is defined as departure from the inpatient ward within 6 h of the decision to admit to the ICU (Table 2). This definition reflects the need to pragmatically distinguish between urgent and elective admission and the recognition that most urgent admission occurs within 1-2 h of the decision to transfer to an ICU.

**Table 3:** **EPOCH secondary outcomes**

**Clinical**	
(1) Significant clinical deterioration event	See Table 4
(2) The nature of clinical deterioration events	Clinical deterioration events will be described by using the Children’s Resuscitation Intensity Scale (Table 4). Urgent PICU admissions that are initiated when the patient is in the operating room will not be regarded as clinical deterioration events
(3) Potentially preventable cardiac arrest	Assessment of the potential preventability of cardiac arrest will be determined for all patients who had a cardiac arrest event while in an eligible inpatient ward, without a preceding DNR order (Table 5)
	Thus, potential preventability ratings of 4: ‘more than likely (more than 50/50, but “close call”);’ 5: ‘strong evidence of preventability;’ 6: ‘virtually certain evidence of preventability’ will be deemed potentially preventable cardiac arrest events
	Preventability will be rated by blinded reviewers reviewing anonymized and delinked clinical data presented in a standardized format. If consensus between the two initial reviewers still cannot be reached then the opinion of the third reviewer will be used as the consensus rating
(4) Unplanned re-admission to the hospital within 48 h of hospital discharge	This outcome will be operationalized as re-admission before midnight of the second day full day after discharge. Thus, re-admission will occur before the 3rd midnight following hospital discharge
(5) Unplanned PICU readmission within 2 days of PICU discharge	This outcome will be operationalized as re-admission before midnight of the second full day after discharge. Thus, re-admission will occur before the 3rd midnight following PICU discharge
(6) PIM score predicted the risk of mortality	
(7) PICU mortality.	
(8) The PELOD score for PICU stay and the first 24 h in PICU	This score will be determined for both the entire PICU stay and the first 24 h in the PICU
(9) Ventilator-free days	Days alive and without invasive mechanical ventilation in the 28 days beginning at PICU admission will be recorded for the first PICU admission during each of the baseline and the post-randomization periods
Process of care	
(1) ‘Stat’ calls to physicians	Requests for immediate specific physician attendance to provide patient care to a patient admitted to an inpatient ward
(2) Code Blue calls	Immediate medical assistance of the resuscitation team and equipment
(3) Urgent consultations to the ICU or MET-RRT	The total number of new consultation episodes will be counted. Patients who have been previously consulted on will be regarded as having a new consult if an urgent call is made that results in an unplanned or earlier than planned review. Planned review involves visits by the ICU team or the MET-RRT
(4) Documentation	The frequency with which each of the ‘vital’ signs (HR, RR, SBP, temperature) and the other four signs of the Bedside PEWS score (transcutaneous oxygen saturation, respiratory effort, oxygen therapy, capillary refill) is documented in 24 h will be recorded from five randomly selected patients each week
Resource utilization	
Hospital length of stay	Will be assessed as the number of patient discharges divided by the number of patient days
following urgent ICU admission	
ICU length of stay	This will be expressed as the number of whole or part study days (00:00:00 – 23:59:59) a given patient was in the ICU
Ventilator days	This is the number of whole or part study days of invasive mechanical ventilation
Dialysis	‘Dialysis’ will include hemo-filtration and hemodialysis techniques used either intermittently and continuously (or both), peritoneal dialysis, plasmaphersis and red-cell exchange
ECMO (days)	This is the number of whole or part study days of extracorporeal membrane oxygenation therapy provided during the ICU stay
Days with nitric oxide	This is the number of whole or part study days of inhaled nitric oxide therapy provided during the ICU stay
Perceptions of healthcare professionals	
Documentation and interaction survey	A 10-min survey of frontline healthcare professionals to describe their perceptions of the utility of the current documentation system, the nature of inter-professional interactions and their background
Decision Maker Study exit survey	Eligible decision-makers will include: hospital chief executive officers (CEOs), chief nursing officers (CNOs), vice presidents and heads of a clinical department, divisions or services. Eligible services include senior nursing administrators for inpatient ward areas, resuscitation committee heads and medical emergency team leaders. At each hospital a maximum of ten eligible leaders will be selected by the EPOCH study team
	A minimum of four decision-makers will be identified: the CEO, CNO, clinical head of pediatric surgery and clinical head of pediatric medicine. Hospitals with more than 80 beds will identify 2 additional decision-makers; hospitals with more than 120 beds will identify 4 additional decision-makers, and hospitals with more than 180 beds will identify an additional 6 decision-makers

**Table 4:** **Clinical deterioration events**

		**Definition**	SCDE*
1	Early transfer	<60 ml/kg intravenous or intraosseous fluid resuscitation given in the 12 h before transfer, no intravenous or intraosseous inotrope or vasoactive medications and no positive pressure ventilation (bag mask or endotracheal) in the 12 h before transfer	No
2	Noninvasive respiratory support	Positive pressure ventilation in the 12 h before transfer, but not intubated at the time of transfer. This category includes children receiving mask-delivered positive airway pressure at any stage in the 12 h before transfer and at the time of transfer. Mechanical ventilation during anesthesia for a scheduled procedure is not included	No
3	Invasive respiratory support	Intubated and/or receiving endotracheal ventilation at the time of transfer or intubated within 1 h of PICU admission	Yes
4	Circulatory	>60 ml/kg intravenous or intraosseous fluid resuscitation given in the 12 h before transfer, and administration of any intravenous or intraosseous inotrope or vasopressor at the time of transfer or at any stage in the 12 h preceding transfer. Patients in this category may also receive positive pressure ventilation (2)	Yes
5	Late transfer	Respiratory (3) *and* circulatory (4) support before transfer	Yes
6	Cardiopulmonary resuscitation	Chest compressions before transfer from ward area or within 1 h of PICU admission or ECMO instituted before or within 1 h of PICU admission	Yes
7	Death	Death on an inpatient ward, other than in those patients with DNR orders. Death may occur despite CPR (or intention to perform CPR if patient is pronounced dead without CPR). No transfer from ward area	Yes

*ECMO* extracorporeal membrane oxygenation therapy.

*SCDE significant clinical deterioration event.

**Table 5:** **Potential preventability criteria**

**Rating**	**Category**
1	Virtually no evidence of preventability
2	Slight-to-modest evidence of preventability
3	Preventability not quite likely (less than 50/50, but “close call”)
4	Preventability more than likely (more than 50/50, but “close call”)
5	Strong evidence of preventability
6	Virtually certain evidence of preventability

Criteria used to determine potential preventability in EPOCH. These criteria were used in the Canadian Adverse Events Study. A rating of 4 or more will be regarded as a high degree of preventability. Potentially preventable cardiac arrests will be presented as rate per thousand patient-days.

### Secondary outcomes: process of care

The study includes five processes of care assessments: (1) ‘Stat’ calls. This was formally defined (Table 3), and a consistent approach to data collection was applied throughout the study, recognizing that cultural and other factors may contribute to considerable inter-hospital variability. (2) Code Blue calls for immediate medical assistance of the resuscitation team and equipment. (3) Urgent consultations (within 15 min) to the ICU or MET-RRT. (4) Using documentation from five randomly (central computerized randomization) selected patients each week, physician visits, the nurse:patient ratio and the use of continuous monitoring are described. (5) Frontline staff complete the documentation and interaction survey (2 pages, <10 min to complete) that describes their perceptions of the documentation system and the nature of interactions with physicians (Additional file 2: CRF). This custom-built survey was developed and used in our pilot evaluations of BedsidePEWS. It has not been formally validated.

### Secondary outcomes: resource utilization and decision-maker perceptions

Resource utilization is assessed in all hospitals by measurement of hospital length of stay and for patients undergoing urgent ICU admission, ICU length of stay and the days of use of the ICU technologies: mechanical ventilation, hemodialysis, ECMO and nitric oxide. A purpose-built survey of the perceptions of hospital decision-makers will be conducted 3 months after the end of the 12-month intervention period (Table 3) to describe the experience of the decision-maker in participating in a hospital-wide cluster RCT and their expectations of its results.

All outcomes are assessed prospectively. The first 26 weeks are baseline data. Then following a 5-week run-in period in intervention hospitals or break from data collection in control hospitals, outcomes are assessed prospectively for 52 weeks (Additional file 1).

### Sample size and assumptions

Power calculations are based on population estimates derived from 14 local hospitals’ data using a published method for cluster RCTs [61]. For all-cause mortality we found a baseline rate of 5.1/1000. The steering committee agreed that a mortality reduction of less than 1/1000 admissions would not be a compelling reason to modify practice. A study with 20 hospitals can show an 18% relative risk reduction in mortality (absolute risk reduction 0.09%), given alpha = 0.05 (2-sided), power = 80%, mean of 119 pediatric beds, length of stay = 4 days, with 0.90 average occupancy, k = 0.15, *n* = 20, baseline rate = 5.1/1000. The quantity k is the coefficient of variation for mortality between hospitals. Assuming attrition of 1-2 hospitals, we will include a sample of 22 hospitals.

Data from 4 pediatric hospitals in Ontario indicate there are 1052 urgent ICU admissions/year, of which we estimate 40% involve SCDE, for a rate of 2 per thousand patient days. Thus, with 20 sites we will be able to show a 31% reduction in SCDE [alpha = 0.05 (2-sided), power = 80%, k = 0.15, where k is the coefficient of variation for SCDE rate between hospitals]. The value of k allows for a moderate amount of clustering within hospital.

### Data analysis

Demographic and unadjusted outcomes data will be reported using means, median, variances, interquartile ranges or as proportions with 95% confidence intervals. Outcomes will be reported for the baseline and intervention periods for each hospital consistent with published recommendations for cluster randomized trials [62].

#### Primary analysis

All-cause hospital mortality will be evaluated using a logit regression model. The dependent variable will be the logit of the proportion dying in each hospital. The independent variables will include a dummy indicator for treatment arm, the baseline mortality logit and the hospital size stratification variable. The analysis will be weighted by the size of the hospital.

#### Secondary analyses

An identical logit model will be used for ICU mortality after urgent ICU admission where the PICU admission was initiated in a hospital ward, ICU mortality after urgent ICU admission initiated in the OR, unplanned hospital re-admission within 2 days of hospital discharge and mortality following DNR orders.

Significant clinical deterioration events, Code Blue events, stat calls and urgent PICU consultations per 1000 patient-days will be evaluated using Poisson regression using hospital-level aggregated count data. The independent variables will include a dummy indicator for treatment arm, the baseline event rate and hospital size stratification variable. A linear regression model, in which the within-hospital mean is the dependent variable, will be used to evaluate the nature of SCDE, inpatient ward patient-days, and for patients admitted urgently to the PICU, PIM2 score, PELOD score, ventilator-free days, ICU patient-days and ICU therapy-days. The independent variables will include a dummy indicator for treatment arm, the baseline means and hospital size stratification variable. The analysis will be weighted by the size of the hospital.

An identical linear regression model will be used to evaluate the frequency of vital sign documentation from the randomly selected patient records. Since the number of records abstracted will be the same in each hospital, weighting will not be necessary. Documentation will be evaluated as follows: the number of heart rate, respiratory rate, systolic blood pressure, trans-cutaneous oxygen saturation, capillary refill, oxygen therapy and respiratory effort measurements, and the number of times that all seven items were documented will be compared for patients in hospitals with and without BedsidePEWS. All analyses will adjust for baseline event rates and hospital strata. Linear regression will be used to compare the numbers of (1) vital signs documented, (2) documented physician visits, (3) the nurse: patient ratio, the use of (4) continuous ECG monitoring and (5) continuous pulse oximeter monitoring with the BedsidePEWS scores calculated from the abstracted clinical data.

Analysis of the documentation and interaction survey and the post-study decision-maker survey data will compare groups using linear regression weighted by the size of the hospital.

The following four pre-specified subgroup analyses will be performed. (1) Hospital size. Hospitals will be classified on the basis of the number of eligible inpatient ward beds. Hospitals with ≥200 eligible inpatient ward beds will constitute one group and those with <200 eligible inpatient ward beds the other. This is consistent with the stratification method used for randomization. (2) Hospitals with and without MET-RRT. (3) Hospitals with ECMO for children. (4) Patients with urgent PICU admission initiated in an inpatient ward.

### Planned conduct

Initial application for funding was made in September 2009, accompanied by 18 letters from hospital CEOs or other senior administrators indicating willingness for their hospital to participate. Successful re-submission in March of 2010 preceded announcement of funding in July 2010. Study resources were obtained from Canadian Institutes of Health Research (CIHR). After approval of $4 million by the reviewing panel, a budget of $3.3 million was granted. Funds were released by the CIHR in December 2010 following recruitment and approval of the three-person data safety and monitoring board, clinical trial registration (CTN#1000018562) and Research Ethics Board (REB-IRB) approval at the sponsoring institution. The study coordinating center is at the Center for Safety Research at the Hospital for Sick Children, Toronto. The study staff is supported by the infrastructure of the Child Health Evaluative Sciences Program of the Research Institute of the Hospital for Sick Children.

EPOCH REB-IRB applications sought and obtained waiver of patient-level consent (Table 6). The local documentation system is used as a part of routine hospital practice, patient-level data are retrospectively obtained and precedent exists in Canada and the UK [51,52]. Furthermore, consent for the participation of 100,000 patents is not feasible, and if required, would significantly undermine the scientific validity of the study. REB-IRB applications also seek approval of implied consent from healthcare professionals for staff surveys and the post-study survey of administrative perspectives [52]. Hospital recruitment though the networks of the CCCTG preceded the enrollment of hospitals with an identified site investigator, administrative and research ethics board approval, and it was contingent on the number of sites previously enrolled in the study. The enrolled sites and associated ethical approvals are provided in Additional file 2.

**Table 6:** **Rationale for waived patient consent in EPOCH**

1	Consent for routine documentation practice is implied with hospital admission + this is also true for other practices including staffing, ICU consultation, physician review
	+ Documentation is an inherent and routine part of hospital care
	+ In intervention hospitals the BedsidePEWS becomes the accepted standard for documentation
	+ In control hospitals and before implementation in hospitals randomized to implement BedsidePEWS, consenting to routine care in a situation where that there is no alternative is counter-intuitive
2	Patient-level data are retrospectively obtained and + does not require patient contact
	+ does not require additional clinical investigation
	+ precedent exists for waived consent for this type of data collection
3	Preemptive consent for events (including in-hospital cardiac arrest, death) that have not occurred - and that may not occur
	+ Is potentially distressing to families
	+ Is inefficient use of research resources
4	Obtaining consent is not feasible for 100,000 patients anticipated in the study sample
	+ retrospective consent from families of deceased children may add burden and is potentially distressing to families
5	Incomplete enrollment would undermine and bias the scientific validity of the study
6	Data will be presented in aggregate.
	No identifying information will leave the study office in the participating hospital

### Conflict of interest management

There are two parties with conflict of interest that are involved in EPOCH. These are the PI (CSP) and the host institution, The Hospital for Sick Children, who are both shareholders in a computerized decision support company (Bedside Clinical Systems). BedsidePEWS is an FDA-approved product of Bedside Clinical Systems. The study PI (CSP) is a named inventor of the BedsidePEWS (US patent 12/669,896). The patent is owned by the host institution (SickKids).

Study processes to ensure transparency, independence and oversight are in place to manage these conflicts of interest. First, EPOCH employs routine quality assurance processes including Data Safety Monitoring Board Review, annual reporting to CIHR, and scientific and operational oversight from the Executive Steering Committee and the Canadian Critical Care Trials Group (CCCTG).

Second, the PI is separated from key study processes including randomization, allocation concealment, data inspection and the conduct of analyses. The study statistician (AW) is responsible for overseeing the random allocation of sites to intervention or control arms, supervises the project manager and uses a password-protected account on randomizedtrials.net. The password is known only to the project manager and statistician, who complete the randomization process and inform the PI and site investigators of the outcome during the first 2 weeks of data collection at each site.

Third, review of site progress in sites randomized to implementation of the BedsidePEWS is conducted by the Executive Steering Committee in the 4th week of the run-in phase. The decision to complete or prolong the run-in phase is made by the majority vote of the Executive Steering Committee. The PI does not vote.

Fourth, data integrity is ensured by three methods. (1) Separation of data collection from randomization status will be achieved by training site coordinators before randomization, using standardized materials. (2) Inspection of study data during site visits is conducted by the Center for Safety Research staff. If present, the PI will not review primary study data. (3) Following creation of the ‘locked’ data set, each site investigator receives a data report to review, reconcile with the data from their site and provide written confirmation that the data are correct or require reconciliation. Once confirmed by all site investigators the final data set will undergo planned analyses.

Fifth, once completed the analyses will be reviewed by an independent statistician based at another university.

Sixth, the interpretation of the data and their reporting in iterative drafts of the main study manuscript will be a shared activity of the executive steering committee and writing committee and will be complimented by pre-submission review by independent CCCTG reviewers.

## Discussion

Preliminary data suggest that the BedsidePEWS score can identify patients at risk for near and actual cardiopulmonary arrest, can be calculated both rapidly and reliably and that its clinical implementation is associated with potential improvements in patient and process of care outcomes. The resource implications of system-wide implementation, the previous lessons from the MERIT study, and the uncertain effect on important clinical outcomes underscore the relevance of the cluster-randomized design, the selection of the intervention and the outcomes used in EPOCH. EPOCH will be conducted in 22 hospitals, involve an estimated 100,000 patients admitted for about 400,000 patient-days and describe approximately 1900 urgent ICU admissions, 200 cardiac arrest events and 500 deaths. Mortality in hospitalized children is an objective measure of quality of care and is used extensively to benchmark the performance of PICUs [58,63-66], surgical programs and hospitals [67-69].

All-cause hospital mortality was selected as the primary outcome in EPOCH for the following reasons. First, mortality is objective and can be reliably assessed. Second, a reduction in all-cause hospital mortality is the ultimate goal of any intervention to improve the outcomes of care of hospitalized children. Third, observational studies of MET-RRT have reported reduced all-cause mortality [36,38]. Fourth, several studies show that when children who have been hospitalized die, most do so in hospital [70-73]. In a 6-year study of in-ICU cardiac arrest, we found only one (1.4%) additional death after index-hospitalization discharge and within 12 months of the index cardiac arrest. Fifth, the effect of pediatric palliative care services on displacing place of death to home or hospice is small [72,74-77]. One large US study found the proportion of deaths in the hospital decreased by less than 6% (from 85.7 to 80.1%) over a 10-year period [75]. Thus, measuring hospital mortality provides a reasonably accurate measure of mortality in children who have been or are currently hospitalized.

As well, all-cause mortality is an established quality metric in Canadian and British adult hospitals and is publicly reported as the standardized mortality ratio in Ontario Hospitals [68,69,78-80]. Finally, while death ‘with DNR’ vs. ‘unexpected’ deaths (no DNR) has been used to distinguish ‘preventable’ from ‘unpreventable’ deaths following acute events in adult patients [34,81], in hospitalized children there are significant limitations to this distinction: (1) DNR orders reflect current expectations of outcome and not the ‘preventability’ of the preceding clinical events; (2) the majority of deaths in hospitalized children, especially in the ICU, occur some days after a clinical deterioration event [3,7,10,18,21,82], but within a relatively short time (hours) between DNR order and death [83-85]. Before confirming that a death in a patient with a DNR order was truly unpreventable, the clinical context and timeline of clinical deterioration prior to the DNR order must be known. It must also be recognized that medically ‘futile’ resuscitation may be performed on hospitalized children [86] for whom no DNR order is in place.

EPOCH outcomes are assessed for 1 year after hospital-wide implementation of intervention or continuation of standard care. This duration was chosen to reduce the effects of seasonal variation, but also to reduce the burden of a multi-year intervention period on each hospital, thus increasing feasibility, and to support timely completion of the study to support the decision-making needs of administrators. Continued eligibility will be confirmed by intermittent reporting of the MET-RRT and Early Warning Score use during the course of the study.

### Limitations of EPOCH

EPOCH has some limitations. First, by excluding the use other severity of illness scores, EPOCH will not provide data comparing alternate scores. This more refined question will need to be evaluated separately, preceded by appropriate studies to support selection of best comparators. Evaluation of the ‘best available’ system is important for proof of concept, needed before these more refined questions are addressed. Second, EPOCH does not assess quality of life, parental perceptions or neuro-cognitive outcome. All are relevant measures of the process and outcome of care [11]. In other prospective work we have shown that urgent ICU admission is associated with reduced health-related quality of life and acquired neuro-cognitive deficits [11]; thus, it would be reasonable to include these measures as outcomes in EPOCH. Exclusion of these elements was due to logistic constraints; acquisition of these data for all admissions would not be feasible, and following these urgent ICU admissions would be both resource intense and would require prospective consent/assent and follow-up. Finally, mortality may be both too coarse a measure and too infrequent an event to demonstrate clinically important differences.

EPOCH is a 22-hospital cluster-randomized trial evaluating the impact of the BedsidePEWS as a mechanism to improve the identification of children with evolving critical illness, reduce near and actual cardiopulmonary arrest, and reduce all-cause hospital mortality in hospitalized children. The results of this trial will provide a scientific basis for local, regional, provincial and national decision-making and for recommendations to national and international bodies [87], such as the AHA and European Resuscitation Council [88] about cardiac arrest prevention and institutional best practices.

If negative, the costs of hospital-wide implementation can be avoided, resources more appropriately allocated and the collaborative multi-site data set from the study used to develop and evaluate new hypotheses about cardiac arrest prevention and processes of care. If positive, then EPOCH will have provided a scientific justification for the major system-level changes required for implementation and supported the creation of evidence-based policy.

### Trial status

At time of manuscript submission (October 2014) ethical approvals had been obtained at each participating site, study enrolment was complete, data collection had ended in 19 hospitals and was anticipated to be completed by July 2015 in remaining sites.

## Additional files

Additional file 1:
**EPOCH study flow diagram. EPOCH timelines and study flow diagram.** Three periods are described: a 26-week baseline period, a 5-week run-in phase during which hospitals randomized to implement BedsidePEWS can become familiar with the implementation and a 52-week post-randomization phase. The blue stars represent the administration of the Documentation and Interaction Survey, and the ‘Q’ represents administration of the decision-maker survey. Randomization occurs in the second week of data collection, and allocation is revealed to the primary investigator and relevant site investigator during the second study week. Additional file 2:
**We list the participating Hospital, the approving Approving Research Committee and the associated reference number for participating sites in the EPOCH cluster randomized trial.**

## Abbreviations

AHA: American Heart Association
BedsidePEWS: Bedside Paediatric Early Warning System
CCCTG: Canadian Critical Care Trials Group
CIHR: Canadian Institutes of Health Research
DNR: Do-not-resuscitate order
EPOCH: Evaluation of processes of care and the outcomes of children in hospital
ICU: Intensive care unit
MET-RRT: Medical emergency team - rapid response team
