# Supplementary figures and images for: Evaluating processes of care and outcomes of children in hospital (EPOCH): study protocol for a randomized controlled trial
Source: Trials. 2015 Jun 2;16:245. doi: 10.1186/s13063-015-0712-3 (PMC4458338; doi:10.1186/s13063-015-0712-3)

Figure 1

# Project timelines, study measurements & personnel

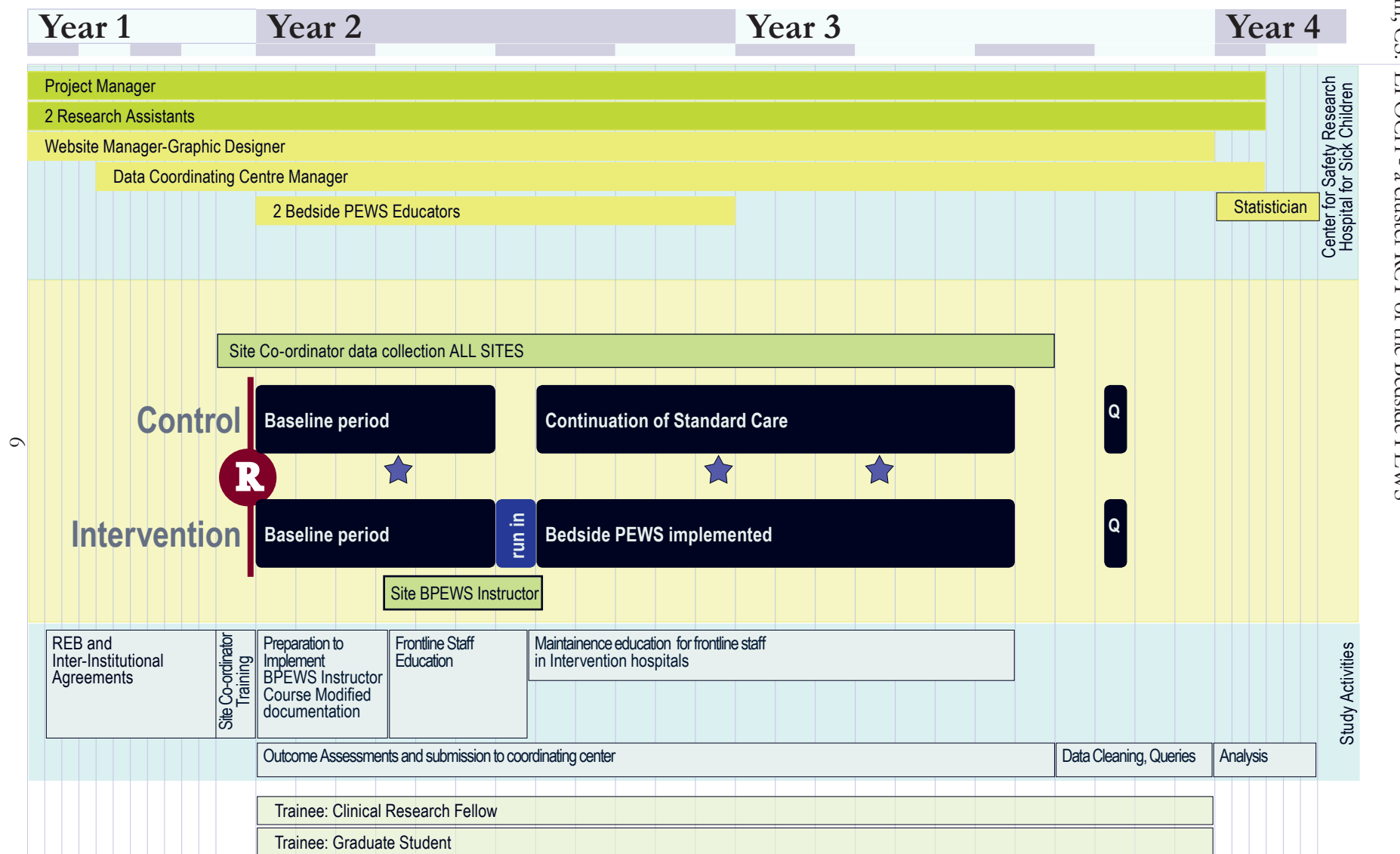

Supplement: Additional file 1: — EPOCH study flow diagram. EPOCH timelines and study flow diagram. Three periods are described: a 26-week baseline period, a 5-week run-in phase during which hospitals randomized to implement BedsidePEWS can become familiar with the implementation and a 52-week post-randomization phase. The blue stars represent the administration of the Documentation and Interaction Survey, and the ‘Q’ represents administration of the decision-maker survey. Randomization occurs in the second week of data collection, and allocation is revealed to the primary investigator and relevant site investigator during the second study week. [file 13063_2015_712_MOESM1_ESM.pdf]
