# Supplementary material for: Evaluating processes of care and outcomes of children in hospital (EPOCH): study protocol for a randomized controlled trial
Source: Trials. 2015 Jun 2;16:245. doi: 10.1186/s13063-015-0712-3 (PMC4458338; doi:10.1186/s13063-015-0712-3)
Supplement: Additional file 2: — We list the participating Hospital, the approving Approving Research Committee and the associated reference number for participating sites in the EPOCH cluster randomized trial. [file 13063_2015_712_MOESM2_ESM.docx]

## Additional file 2

| Hospital Name | Approving Research Committee Name | Reference |
| --- | --- | --- |
| Hospital for Sick Children | Research Ethics Board Hospital for Sick Children | 1000018562 |
| Starship Hospital | Northern X Regional Ethics Committee | NTX/11/02/002 |
| Saint John Regional Hospital | Research Services - Atlantic Health Sciences Corporation | NCT0126083 |
| IWK Childrens Hospital | IWK Health Centre - Research | 1006406 |
| Montreal Childrens Hospital | The Research Institute of the McGill University Health Center | 11-502-PED |
| Royal St Georges Hospital | National Health Service Research Ethics Committee | 11/LO/0502 |
| Kings College Hospital | National Health Service Research Ethics Committee | 11/LO/0502 |
| The Royal London Hospital | National Health Service Research Ethics Committee | 11/LO/0502 |
| Royal Brompton Hospital | National Health Service Research Ethics Committee | 11/LO/0502 |
| St Mary's Imperial Hospital | National Health Service Research Ethics Committee | 11/LO/0502 |
|  |  |  |
| Le Centre hospitalier universitaire de Québec | CHU de Quebec Research Center | C11-03-177-21 |
| St JustineS Hospital | Research Ethics Board of the Research Centre of CHU Sainte-Justine | 3338 |
| Stollery Childrens Hospital | Health Research Ethics Board | MS3_Pro00020320 |
| Victoria General Hospital | Vancouver Island Health Authority- Heath Research Ethics Board | H2011-52 |
| McMaster Childrens Hospital | Hamilton Health Science - REB | 11-281 |
| London Health Sciences Centre | Lawson Health Research Institute | 18470 |
|  |  |  |
| Alberta Childrens Hospital | Office of Medical Bioethics - University of Calgary | E-23968 |
| Children's University Hospital | Children's University Hosptial Ethics Committee | 11.019 |
| Our Lady's Children Hospital | Our Lady's Children Hospital for Sick Children Ethics Committee | NA |
|  |  |  |
| Bambino Gesu Ospedale Pediatrico | Instituto di Ricovero e Cura a Caratterre Scientifico | NCT01260831 |
